# Tuning the permselectivity of polymeric desalination membranes via control of polymer crystallite size

**DOI:** 10.1038/s41467-019-10132-0

**Published:** 2019-05-28

**Authors:** Xinglin Lu, Xunda Feng, Yi Yang, Jin Jiang, Wei Cheng, Caihong Liu, Manesh Gopinadhan, Chinedum O. Osuji, Jun Ma, Menachem Elimelech

**Affiliations:** 1 Department of Chemical and Environmental Engineering, Yale University, New Haven, CT 06520-8286 USA; 2 State Key Laboratory of Urban Water Resource and Environment, Harbin Institute of Technology, Harbin, 150090 China; 3 Center for Advanced Low-dimension Materials, State Key Laboratory for Modification of Chemical Fibers and Polymer Materials, Donghua University, Shanghai, 201620 China

**Keywords:** Chemical engineering, Polymer characterization, Polymers

## Abstract

Membrane desalination is a leading technology for treating saline waters to augment fresh water supply. The need for high-performance desalination membranes, particularly with high water/salt selectivity, has stimulated research into the fundamental structure-property-performance relationship of state-of-the-art membranes. In this study, we utilize a facile method for tuning properties of a polymeric desalination membrane to shed light on water and salt transport mechanisms of such membranes. A desalination membrane made of cellulose triacetate is treated in a plasticizer solution, followed by water rinsing. The modified membranes exhibit reduced salt flux without compromising water flux, indicating enhanced water/salt selectivity. An inspection of material characteristics using a model film system reveals a plasticizing-extracting process in changing the polymeric structure, which leads to the reduction of crystallite size in the polymer matrix, consequently affecting the transport properties of the membranes. Our findings highlight the potential of the plasticizing-extracting process in fabricating membranes with desired desalination performance.

## Abstract (web summary)

Increasing the selectivity of desalination membranes is of crucial importance for advancing membrane desalination technologies. Here the authors demonstrate a plasticizing-extracting process for tuning the permselectivity of polymeric membranes and achieving desired desalination performance.

## Introduction

Access to clean, safe, and adequate water supply is a human right^1^, essential for humanity’s sustainability and well-being^2^. However, rapid population growth and industrialization, as well as the associated water resource contamination, have led to the reality that over one billion people around the globe face water shortages^3^. This global crisis motivates technological innovations for water purification. Given the progress in membrane science in the last few decades, membrane desalination has become a leading technology in alleviating water scarcity through treating saline waters (i.e., seawater and brackish water), the most abundant water resources on Earth^4,5^. Currently, reverse osmosis (RO), as the most energy-efficient desalination technology^5^, is daily augmenting over 20 million cubic meters of fresh water to millions of people living in water-stressed regions^6^.

The development of membrane desalination technologies relies on advances in membrane materials^7,8^. State-of-the-art commercial products are polymeric membranes^7^, either cellulose-based asymmetric type or polyamide thin-film composite type, which were successfully fabricated several decades ago^9,10^. Although some emerging materials, such as nanotubes^11,12^, two-dimensional nanosheets^13,14^, and aquaporins^15,16^, have been extensively researched as candidates for next-generation desalination membranes, their real-world applications are still hindered by low separation performance and poor long-term stability^17^. In the foreseeable future, polymeric membranes will likely retain their dominant roles in the global desalination market^18,19^. Therefore, enhancing the performance of existing polymeric membranes is more practical for improving the system efficiency of current desalination systems. Such enhanced performance requires a deeper understanding of the fundamental structure-property-performance relationship^8^.

Water and salt transport in dense polymeric membranes is governed by the solution-diffusion mechanism, where small molecules first partition into the polymer matrix and then diffuse to the permeate side under a chemical potential gradient^20,21^. This mass transport phenomenon takes place within the free volume of amorphous regions, that is, a segment of the polymer matrix with high mobility of polymeric chains^22–25^. Previous studies have demonstrated that tuning the properties of free volume, either through the induction of chain mobility or rearrangement, could impact the permselectivity of gas separation membranes^26,27^ or ion-exchange membranes^28,29^, whose transport behaviors are also governed by the solution-diffusion mechanism. However, the actual effect of polymer structure on water and salt transport in desalination membranes has not been well documented. Given the need for fabricating desalination membranes with increased selectivity^8,30,31^, it is critical to understand the role of polymer structure in desalination performance to guide membrane design.

Here, we demonstrate a facile method to tune the transport properties of a polymeric desalination membrane via a plasticizer-induced swelling and subsequent deswelling process, which results in modification of the internal structure of the polymer material. Compared with the pristine membrane, the modified membrane displays significantly enhanced water/salt selectivity and desalination performance. Investigations of material characteristics reveal that the plasticizer, p-nitrophenol, penetrates the polymeric matrix and transforms crystalline regions to amorphous regions. Subsequent extraction of the plasticizer from the polymer matrix by water rinsing induces the rearrangement of polymer chains followed by recrystallization. Moreover, structural characterization by optical microscopy and X-ray diffraction suggests a size reduction of the crystallites distributed in the amorphous matrix where the latter primarily governs the mass transport properties. We propose that the reduced crystallite size results in an enlargement of the interfacial area between the crystalline and amorphous regions, which is responsible for the observed enhanced water/salt selectivity. Our findings demonstrate that the plasticizing–extracting process can be harnessed to improve the desalination performance of asymmetric polymeric membranes.

## Results

### Effect of PNP treatment on membrane transport properties

**Fig. 1:**
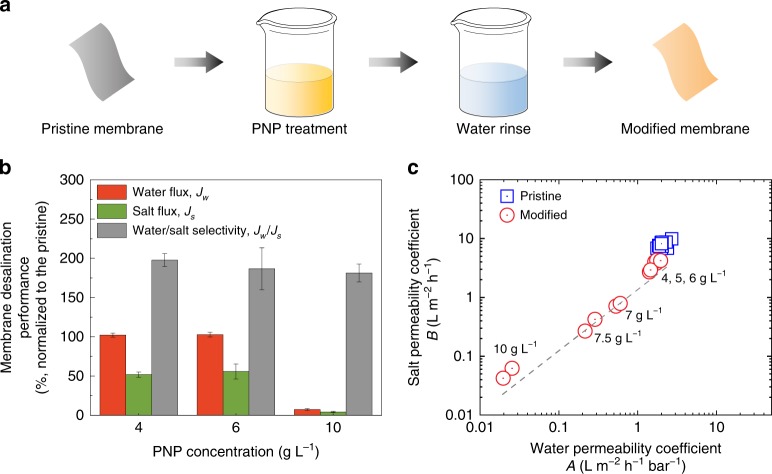
Enhanced desalination performance and water/salt selectivity of the polymeric membrane through p-nitrophenol (PNP) treatment. **a** Schematic illustration of PNP treatment procedure. A pristine CTA membrane is soaked in a PNP solution, followed by a thorough rinse with water to obtain a modified membrane. **b** Influence of PNP modification on desalination performance, including water flux, salt flux, and water/salt selectivity (ratio of water flux to salt flux). The pristine CTA membranes were soaked in PNP solution at various concentrations at pH 4.0 for 0.5 h. The soaked membranes were subsequently rinsed using water for 8 h. Membrane performance results were normalized to that of the corresponding pristine membranes. Error bars represent standard deviation from duplicate experiments. **c** Correlation between water permeability coefficient, *A*, and salt permeability coefficient, *B*, for CTA membranes modified at different PNP concentrations. The dashed line is an eye guide showing the change of transport properties with an increase of PNP concentration

We developed a simple procedure to tune the performance of polymeric desalination membranes, as schematically illustrated in Fig. 1a. Briefly, pristine cellulose triacetate (CTA) membranes were soaked in a p-nitrophenol (PNP) solution, followed by water rinsing to obtain modified membranes. The membranes were subsequently characterized in a forward osmosis (FO) setup to evaluate the effect of PNP treatment on desalination performance, namely water flux, *J*_w_, and reverse salt flux, *J*_s_. Additionally, we calculated the ratio of *J*_w_ over *J*_s_, which is expressed as^32,33^

1 $$\frac{{J_{\mathrm{w}}}}{{J_{\mathrm{s}}}} = \frac{A}{B}nR_{\mathrm{g}}T$$

where *A* and *B* are water and salt permeability coefficients, respectively, *n* is the number of dissolved species created by the draw solute, *R*_g_ is the ideal gas constant, and *T* is the absolute temperature. As *n*, *R*_g_, and *T* are constant parameters, *J*_w_/*J*_s_ is proportional to *A*/*B*, which is an indicator of the change of water/salt selectivity^32,33^.

Figure 1b presents the significant improvement in desalination performance after treatment with PNP solutions of various concentrations, ranging from 4 to 10 g L^−1^. For example, the membrane treated with 4 g L^−1^ PNP solution exhibited a water flux comparable to that of the pristine membrane, as indicated by the normalized water flux of ~102 ± 2.6% (Fig. 1a). However, the salt flux of the modified membrane showed a sharp decrease of 48.3 ± 3.4%. Consequently, the water/salt selectivity of the treated membrane, as determined by the ratio of water flux over salt flux, showed a two-fold increase compared to that of the pristine membrane. Increasing PNP concentration led to more remarkable effects on desalination performance, as evidenced by the significant decreases in both water and salt fluxes of the membranes treated with 10 g L^−1^ PNP solution. Notably, the modified membranes displayed an obvious tendency to curl toward the active layer (Supplementary Fig. 1). The curliness was more pronounced with an increase of PNP concentration from 4 to 10 g L^−1^, in accordance with the extent of performance change. This structural change strongly suggests that PNP treatment induced an asymmetric effect on the active and the support layers of the membrane, thereby eventually influencing the water and salt transport in the polymeric CTA membrane.

We also investigated the influence of PNP species (protonated vs. deprotonated) on desalination performance of the CTA membrane. The two species were obtained by adjusting the pH of PNP solution according to its pK_a_ of 7.1^34^; the extent of water/salt selectivity change was plotted as a function of pH in Supplementary Fig. 2a. After an identical treatment process, the membranes treated with the protonated PNP (pH < 7.1) showed a 90% increase in selectivity, while the effect of PNP on selectivity was practically eliminated for the membranes treated with the deprotonated PNP (pH > 7.1), in which curling was also not observed (Supplementary Fig. 2b). Moreover, under the same effective concentration of the protonated PNP (i.e., 5 g L^−1^), the membranes treated with different PNP solutions (5 g L^−1^ at pH 4 vs. 10 g L^−1^ at pH 7.1) exhibited comparable changes in desalination performance (Supplementary Fig. 3). Taken together, these results suggest that only the protonated form of PNP was responsible for the changes in membrane structure and desalination performance. The ineffectiveness of the deprotonated PNP in affecting membrane properties is likely ascribed to the electrostatic repulsion and the relatively large hydrated size of the anionic deprotonated species compared to the neutral protonated form, thereby lowering its modification effect by hindering adsorption and penetration into the polymer matrix^35,36^. Additionally, the unlikely formation of hydrogen bond between the deprotonated PNP and the CTA polymer is also a critical factor weakening their interactions (detailed analysis provided in the Supplementary Discussion and Supplementary Fig. 4).

Based on the measured desalination performance data, we calculated the membrane transport coefficients, *A* and *B* (calculation details provided in the Supplementary Methods) and summarized all the data in Supplementary Table 1. As the concentration of PNP was increased, both *A* and *B* coefficients declined (Fig. 1c). Notably, the decrease of salt permeability is more pronounced than that of water permeability (Supplementary Fig. 5), in agreement with the increased water/salt selectivity.

### Material characteristics of CTA films treated with PNP

The water/salt selectivity of the asymmetric CTA membrane is solely determined by the top skin layer, and therefore, the observed enhancement in water/salt selectivity via PNP treatment most likely originates from the structural change of the polymer in the skin layer. To circumvent the possible influence of additives and impurities in the commercial membrane, we fabricated a pure CTA film as a model system via an identical phase separation process for mechanistic analysis.

**Fig. 2:**
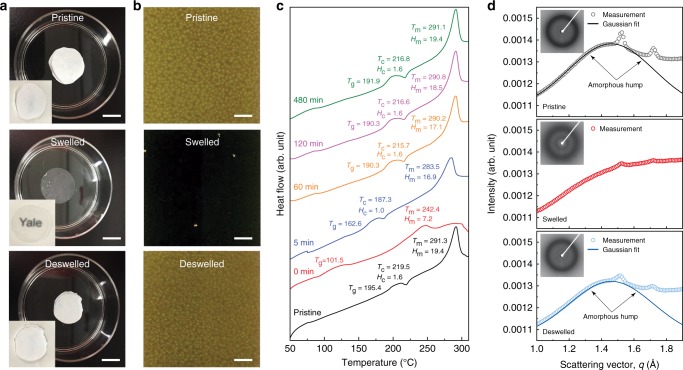
Structural characteristics of CTA films. **a** Digital photos of the CTA films. Scale bar, 1 cm. **b** Polarized optical microscopy images of the CTA films. Scale bar, 200 μm. The pristine film displays whiteness and opaqueness (upper panel in **a**), indicative of the presence of crystalline regions as confirmed by the birefringence (upper panel in **b**). The crystalline structure was destroyed in the swelled film due to the penetration of PNP, which can be demonstrated by the observed transparency (middle panel in **a**) and the loss of the birefringence (middle panel in **b**). Further rinsing with water resulted in the recovery of the white and opaque properties and the birefringence of the deswelled film (lower panels in **a**, **b**), suggesting the recrystallization of the CTA polymer chains. **c** Influence of water rinsing time on the thermal properties of CTA films as determined by differential scanning calorimetry (DSC). After soaking in PNP, the modified membrane without water rinsing (i.e., 0 min) exhibited significant decrease of glass transition temperature (*T*_g_) and disappearance of the cold crystallization peak (i.e., crystallization temperature, *T*_c_, and latent heat of crystallization, *H*_c_) and the melting peak (i.e., melting temperature, *T*_m_, and latent heat of melting, *H*_m_), indicating the solvation of PNP into the polymeric chains. As water rinsing proceeded, both *T*_g_ and crystalline and melting peaks were gradually recovered to values comparable to those of the pristine films, implying that the gradual leaking of PNP induces rearrangement of polymeric chains. **d** One-dimensional X-ray diffraction data displaying the different diffraction peak profiles of the CTA films. Insets are the corresponding 2-D wide-angle X-ray diffraction patterns. The open circles are the measured data and the solid curves are the Gaussian fit of the amorphous hump centered at 1.46 Å^−1^. Generally, the pristine film displayed two strong diffraction peaks (black circles). The diffraction peaks almost vanished after PNP soaking for the swelled sample (red circles). The extraction of PNP from the polymer matrix leads to the recovery of the diffraction peaks of the deswelled sample (blue circles), but the peak widths were broader than those of the pristine film

The as-prepared CTA film (denoted as pristine) contained crystalline micro-domains coexisting with amorphous regions in the polymer, endowing the film with remarkable opaqueness (upper panel in Fig. 2a). This partially crystalline nature of the CTA film can be demonstrated by the birefringence displayed in the polarized optical microscopy (POM) image (upper panel in Fig. 2b). Soaking in a PNP solution, however, significantly changed the optical property of the film (denoted as swelled), as reflected by the observed high transparency of the CTA film (middle panel in Fig. 2a) and the loss of the birefringence in the POM image (middle panel in Fig. 2b). Moreover, the soaked CTA film became soft compared to the pristine one. Such observations suggest a remarkable penetration of PNP into the CTA film that suppressed the crystallinity of the polymer, as soaking the film in pure water did not lead to the observed change. The loss of crystallinity and birefringence implied that PNP acted as a plasticizer in CTA that homogeneously swelled the polymeric chains^37–39^. Since the desalination performance was evaluated when the membrane was thoroughly rinsed by water to release the PNP molecules from the polymer matrix, for consistency, we also rinsed the swelled CTA film with pure water. The washed CTA film (denoted as deswelled, lower panels of Fig. 2a, b) recovered the opaqueness and the birefringence as shown in the pristine film, indicative of a recrystallization process of the CTA polymer following the extraction of PNP. By a careful examination of the POM images (Supplementary Fig. 6), we found a decrease in the size of the birefringent domains in the deswelled film as compared with those of the pristine film. This finding suggests that the average size of the crystallites in the polymer became smaller after PNP treatment.

The plasticizing–extracting process of PNP can be demonstrated by differential scanning calorimetry (DSC) measurements. Figure 2c displays a series of heating curves from DSC measurements of the CTA films with different contents of PNP, which can be controlled by the duration of water rinsing. Note that the films were completely dehydrated before measurements to exclude the possible effects of water. The pristine film had a glass transition temperature (*T*_g_) at 195.4 °C upon heating, cold-crystallized at *T*_c_ = 219.5 °C, and melted at *T*_m_ = 291.3 °C. The swelled film without water rinsing (i.e., 0 min) displayed a sharp drop of the *T*_g_ down to 101.5 °C and almost indistinguishable crystallization and melting peaks, indicative of a strong adverse effect of PNP on CTA crystallization. Release of PNP from the polymer film by water rinsing corresponded to the PNP extracting process, as evidenced by a gradual recovery of *T*_g_ upon increasing the rinsing time in water. For instance, rinsing in water for 5 min resulted in a recovery of *T*_g_ to 162.6 °C. Further increase of the water rinsing time corresponded to elevating the value of *T*_g_; after a prolonged period of water rinsing for 8 h, all the *T*_g_, *T*_c_, and *T*_m_ values were matching to those of the pristine films. Additionally, the 8-h rinsing sample also displayed values of both crystalline enthalpy (*H*_c_) and melting enthalpy (*H*_m_) comparable to those of the pristine sample. Taken together, the essentially unchanged DSC data found in the pristine and the rinsed CTA films suggests that the PNP treatment did not lead to a remarkable alteration of the thermal properties of the polymer.

To elucidate quantitatively why such a PNP plasticizing–extracting process affects the membrane properties, we employed wide-angle X-ray diffraction (WAXD) to characterize the pristine, swelled, and deswelled CTA films, whose 2-D patterns are the insets of Fig. 2d. As seen from the integrated 1-D X-ray diffraction patterns (Fig. 2d), the pristine film has two characteristic diffraction peaks centered at scattering vectors (*q*) of 1.53 and 1.72 Å^−1^, respectively, owing to the presence of crystalline regions in the film. These two peaks almost vanished after the penetration of PNP into the film (swelled sample), indicating the destruction of the crystallized polymer structures by PNP, which is consistent with the loss of the optical birefringence observed in the POM image. Rinsing off the PNP from the films with water (deswelled sample) led to the re-emergence of the two diffraction peaks characteristic of the crystal structure.

The WAXD patterns qualitatively show that the widths of the Bragg scattering peaks in the deswelled film were much broader compared to those of the pristine film. To compare the difference quantitatively, it is necessary to deconvolute the observed diffraction intensity into amorphous and crystalline components^40^. The broad humps centered at *q*_*c*_ = 1.46 Å^−1^ due to the amorphous contribution, as shown in both pristine and deswelled samples, can be well fitted by Gaussian functions as evidenced by R-squared > 99.9% (fitting curves are shown in solid lines in Fig. 2d). The crystalline peak profile was then obtained by subtracting the amorphous component from the original diffraction curve (data processing details appear in the Supplementary Methods). The broadening of the Bragg peaks was then quantified by their full width at half maxima (FWHM). We fitted these peaks using Gaussian functions and found that the peak centered at 1.53 Å^−1^ has an FWHM value of 0.032 and 0.040 Å^−1^ for the pristine and the deswelled samples, respectively (Supplementary Fig. 7). If we ignore any contribution from the instrumental line and microstrains, the broadening of the diffraction peaks indicates the reduction of the crystallite size in the deswelled films. However, the overall crystallinity of the polymer in the deswelled film did not change, consistent with the indiscernible DSC data of the pristine and the deswelled samples.

### Proposed mechanism for the enhanced permselectivity by PNP

**Fig. 3:**
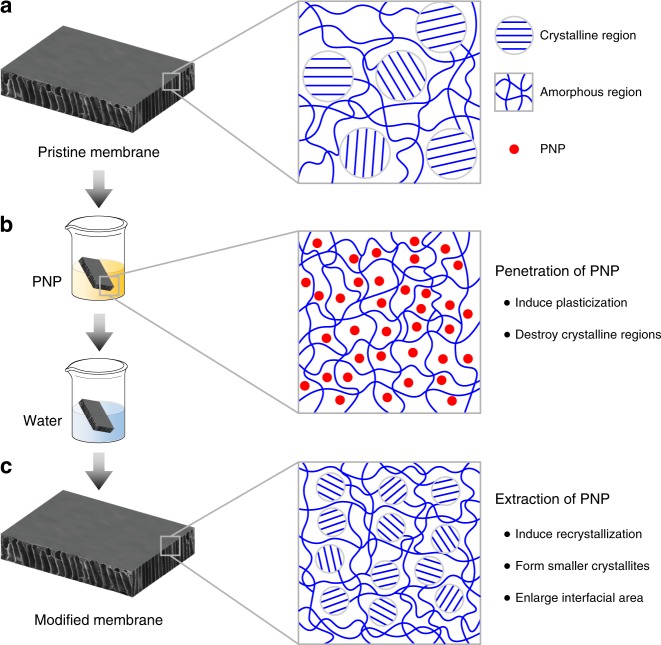
Schematic illustration of a proposed mechanism for the role of p-nitrophenol (PNP) treatment in tuning transport properties of the polymeric desalination membrane. **a** The pristine CTA membrane is semi-crystalline, consisting of large crystallites embedded in amorphous regions. **b** Soaking the membrane in PNP solutions leads to swelling of both the crystalline and amorphous regions by PNP. In this step, PNP acts as a plasticizer to increase the chain mobility of the polymer matrix. **c** Gradual release of PNP by water rinsing results in rearrangement and recrystallization of the polymer chains. This equilibrium process does not affect the overall crystallinity but induces the formation of smaller crystallites, thereby enlarging the interfacial area between the amorphous and crystalline regions. This increased interfacial area could facilitate the fixation of the amorphous loops in the crystalline lattice, thereby reducing the number of nonselective pathways for mass transport

Taken together, the material analysis of the CTA films enabled deeper insight into the impact of PNP on the permselectivity of the CTA membrane. This type of polymeric membrane is fabricated through non-solvent induced phase separation (NIPS), in which extraction of the polymer solvent by a non-solvent induces the precipitation of the polymer. The fast kinetics of NIPS do not allow the CTA polymer chains to undergo crystallization through a slow equilibrium process^21^. Therefore, the majority of the polymeric chains are quenched in the polymer matrix, forming a skin layer comprising large crystallites embedded in amorphous regions (Fig. 3a).

PNP acts as a plasticizer in the CTA polymer (Fig. 3b). Specifically, the partitioning of PNP molecules into the polymeric matrix not only enhances the chain mobility in the amorphous regions but also swells the crystalline domains, resulting in disruption of the molecular packing. This phenomenon was evidenced by the disappearance of crystallinity of the treated membranes through material characterization (POM, DSC, and WAXD). When rinsed with water, the incorporated PNP molecules tend to leach out of the polymer film. The interaction of PNP with the CTA polymer (i.e., hydrogen bonding), together with the relatively low concentration gradient of PNP and the diffusion resistance of the dense polymeric matrix, led to a slow leaching process (demonstrated by the DSC results), which induced an equilibrium rearrangement of polymeric chains to form smaller crystallites (Fig. 3c). Notably, as the overall crystallinity of the deswelled sample did not show a notable change compared to that of the pristine sample, smaller crystallites result in a larger interfacial area between amorphous and crystalline regions. This increased interfacial area could facilitate the fixation of the amorphous loops in the crystalline lattice^41^, thereby reducing the number of nonselective pathways for mass transport, which consequently enhances the water/salt selectivity of the CTA membrane^8,42^.

### Implications for the design of desalination membranes

We demonstrated that the reduction of the crystallite size by the plasticization–extraction of PNP in CTA membranes was the main mechanism for the enhanced water/salt selectivity. While this fundamental analysis was the main goal of this study, the results provide guidelines for the fabrication of membranes with desired performance and permselectivity.

PNP treatment directly influences polymeric membrane transport properties by decreasing both water and salt permeabilities. However, membranes soaked in lower PNP concentrations (e.g., 4 or 6 g L^−1^) exhibited enhanced desalination performance, i.e., a comparable water flux with a lower salt flux. The unchanged water flux is a result of the combined effects of a simultaneous reduction in both water and salt permeabilities. While a lower water permeability leads to a reduction in water flux, a decrease in salt permeability reduces the reverse salt flux, thus reducing internal concentration polarization (ICP) inside the membrane support. In this case, the suppression of ICP, driven by a reduction in reverse salt flux, compensates for the reduction in water flux caused by the reduction in water permeability (details of the mathematical analysis provided in the Supplementary Discussion)^43–45^. Moreover, the enhanced permselectivity, achieved through PNP treatment, is advantageous for improving product water quality in current desalination systems by rejecting pollutants with low molecular weight^30^.

The modified membrane exhibited enhanced water/salt selectivity toward different salts (Supplementary Fig. 8) with long-term stable performance (Supplementary Fig. 9). Optimization of the treatment conditions, such as soaking duration (Supplementary Fig. 10) and type of plasticizers (Supplementary Fig. 11), could lead to further enhancement in desalination performance. The effect of PNP treatment is also observed in different CTA membranes (Supplementary Table 2) and in a different operational mode using RO (Supplementary Fig. 12), suggesting the versatility of the plasticizing–extracting process in improving desalination performance of a variety of polymeric membranes.

In conclusion, we have developed an effective approach to tune the permselectivity of polymeric desalination membranes by a plasticizer-induced swelling and deswelling process. The treated membranes exhibit decreases in both water and salt permeabilities, owing to the reduction of crystallite size in the crystalline regions and chain mobility in the amorphous regions. The more significant effect on salt permeability relative to water permeability led to a higher permselectivity, thereby rendering the modified membrane with enhanced desalination performance. Our findings not only provide mechanistic insights into the structure-property-performance relationship of polymeric desalination membranes, but also offer a lesson for the future design of asymmetric polymeric membranes with desired desalination performance.

## Methods

### Treatment of polymeric CTA membranes using PNP

PNP solutions were prepared by dissolving PNP powder (Sigma-Aldrich) in deionized (DI) water (18.2 MΩ cm, Millipore). The solution pH was adjusted by adding an aliquot of acid (1 M HCl) or base (1 M NaOH). Wet pristine CTA membranes (Hydration Technology Innovation) were soaked in PNP solution at ambient temperature (~20 °C) for 30 min. Following this step, the treated membranes were thoroughly rinsed with DI water to obtain modified membranes. All membranes were stored in DI water at 4 °C before further characterization.

### Characterization of desalination performance

Membrane desalination performance was characterized using a custom-built forward osmosis (FO) setup (Supplementary Fig. 13); details of the experimental procedures are provided in the Supplementary Methods. Briefly, a membrane coupon was sandwiched between draw solution (1 M NaCl) and feed solution (DI water) with the skin layer facing the draw solution. Water flux, *J*_w_, and reverse salt flux, *J*_s_, were obtained by measuring the change of volume and salt concentration in the feed solution using a scale and a conductivity meter, respectively. Considering the inherent variation in membrane performance among coupons, we characterized the desalination performance of every pristine membrane prior to the modification. Results for a modified membrane were normalized to its corresponding pristine data. Based on the measured desalination performance data, we calculated the transport coefficients, including water permeability, *A*, and salt permeability, *B*, which are independent of testing conditions (details in the Supplementary Methods).

### Fabrication of model CTA films

Model CTA films were fabricated through non-solvent induced phase separation (NIPS), which is identical to the process used for commercial CTA membranes. CTA beads (12 wt%) were dissolved in dimethylacetamide (DMAc), stirred for 12 h, and deaerated in a desiccator for at least 15 h prior to casting. A casting knife (Gardco, US), set at a gate height of 250 µm, was used to spread the CTA polymer solution over a glass surface. The cast film was immediately immersed in a precipitation bath containing 3 wt% DMAc in DI water at room temperature (~20 °C) to initiate phase separation. The formed CTA film remained in the precipitation bath for 10 min before being transferred to a water bath for storage. These pristine CTA films also underwent a modification procedure identical to that for the commercial membrane coupons to obtain modified films. The films were air dried prior to material characterization.

### Material characterization of CTA films

Polarized optical microscopy (POM) images were obtained by an inverted microscope (Axiovert 200 M, Zeiss). Differential scanning calorimetry (DSC) measurements were conducted using a Q200 DSC (TA Instruments) at a heating/cooling rate of 20 °C min^−1^. The polymeric film samples were preheated to 100 °C twice to evaporate any residual water or solvent prior to the first heating cycle. Two-dimensional wide-angle X-ray diffraction (2-D WAXD) measurements were performed using a Rigaku 007 HF+ instrument equipped with a rotating anode Cu KR X-ray source (λ = 1.542 Å) and a 2-D Rigaku Saturn 994+ CCD detector. The calibration of the resultant 2-D WAXD patterns was done by using a silver behenate standard (d-spacing of 3.1355 Å). All 2-D X-ray diffraction patterns were integrated into 1-D plots of diffracted intensity (*I*) versus the scattering vector, *q*, where *q* *=* 4πsin(θ)/λ and the diffraction angle is 2θ.

## Supplementary information

Supplementary Information

## Data Availability

The data that support the findings of this study are available from the corresponding authors upon reasonable request.
