# Supplementary material for: Tuning the permselectivity of polymeric desalination membranes via control of polymer crystallite size
Source: Nat Commun. 2019 May 28;10:2347. doi: 10.1038/s41467-019-10132-0 (PMC6538653; doi:10.1038/s41467-019-10132-0)
Supplement: Supplementary file 1 — Supplementary Information [file 41467_2019_10132_MOESM1_ESM.pdf]

Supplementary Information

Tuning the permselectivity of polymeric desalination membranes via control of polymer crystallite size

Lu et al.

Supplementary methods

Materials and chemicals

A commercial desalination membrane made of cellulose triacetate (CTA) was obtained from Hydration Technology Innovation (HTI, USA). This asymmetric membrane is prepared through non-solvent induced phase separation and is widely used in forward osmosis (FO) desalination processes. Another type of denser CTA membrane was also obtained from HTI and used as a comparison. Prior to the experiments, all pristine CTA membranes were cut into 3 cm × 9 cm coupons and immersed in water overnight to allow complete wetting. Unless otherwise specified, all the chemicals were purchased from Sigma-Aldrich and used as received. Deionized (DI) water obtained from a Milli-Q ultrapure water purification system (Millipore, USA) was used in all the experiments.

Characterization of membrane desalination performance in forward osmosis (FO)

A custom-built crossflow FO unit with channel dimensions of 80 mm × 20 mm × 3 mm was used to evaluate desalination performance (i.e., water flux and reverse salt flux) of the membrane. A schematic of the FO setup used is shown in Supplementary Figure 13.

The experiments were performed using 1 mol L^{-1} NaCl draw solution and DI water feed solution, generating a bulk osmotic pressure difference of 48.4 bar (OLI Systems Inc., USA). The membrane is loaded into the cell with the active layer facing the draw solution. Variable speed gear pumps (Longer, China) were used to generate co-current crossflows of 16 cm s^{-1}. Temperatures of both draw and feed solutions were maintained at 25.0 ± 0.5 °C by a water bath (Tianheng Instrument, China).

Water flux, J_w, across the membrane was measured by recording the weight increase of the draw solution at three-minute intervals and calculated by:

$$J_w = \frac{\Delta m/\rho}{At} \tag{1}$$

where Δm is the measured weight change of the draw solution, ρ is the density of water, A is the membrane area, and t is the time.

A conductivity meter probe was immersed in the feed solution side to determine the increase of NaCl concentration in the feed solution at three-minute intervals. Reverse salt flux, J_s, was calculated by:

$$J_{\mathrm{s}} = \frac{C_{\mathrm{F}}(V_{\mathrm{F}}^{\mathrm{Initial}} - J_{\mathrm{w}}At)}{At} \tag{2}$$

where C_{F} is the NaCl concentration in the feed solution, $V_{\mathrm{F}}^{\mathrm{Initial}}$ is the initial volume of the feed solution, A is the membrane area, and t is the time.

Calculation of membrane transport properties

The transport properties of the membranes were determined based on the desalination performance data in FO. In particular, salt permeability, B, was first calculated using[1]:

$$B = \frac{J_{\mathrm{s}} J_{\mathrm{w}} \exp\left(Pe^{\mathrm{S}}\right)}{J_{\mathrm{s}}\left[1 - \exp\left(Pe^{\delta} + Pe^{\mathrm{S}}\right)\right] + J_{\mathrm{w}} C_{\mathrm{D}}} \tag{3}$$

where C_{D} is the concentration of the NaCl draw solution; Pe^{δ} is the Peclet number of the boundary layer in the draw solution side with external concentration polarization (ECP):

$$Pe^{\delta} = \frac{J_{\mathrm{w}}}{k} \tag{4}$$

where k is the feed side mass transfer coefficient, which can be estimated from a correlation for the rectangular cell geometry and given operating conditions. Pe^{S} is the Peclet number of the support layer in the feed side with internal concentration polarization (ICP):

$$Pe^{\mathrm{S}} = J_{\mathrm{w}} \frac{S}{D} \tag{5}$$

where S is the structural parameter of the CTA membrane, which was determined to be ~595 μm according to a previous publication using the same membrane[2]. Then, water permeability, A, was determined using[3,4]:

$$A = \frac{J_{\mathrm{w}} B}{J_{\mathrm{s}} n R_{\mathrm{g}} T} \tag{6}$$

where n is the number of dissolved species created by the draw solute, R_{g} is the ideal gas constant, and T is the absolute temperature. The calculated transport properties of membranes are listed in Supplementary Table 1.

Methodology for deconvoluting X-ray diffraction data into amorphous and crystalline components

Both amorphous and crystalline regions in the cellulose triacetate films contribute to the X-ray diffraction patterns. The diffraction intensity, I, as a function of scattering vector, q, which was obtained from the measurement and can be written as:

$$I(q) = I_A(q) + I_C(q) \quad (7)$$

where $I_A(q)$ and $I_C(q)$ are the amorphous and crystalline contributions, respectively.

In order to analyze the Bragg peak profiles, one must separate the amorphous contribution from the total intensity vs. q. The broad hump centered at q_c= 1.46 Å^{-1} in the 1-D integrated curve, corresponding to the amorphous halo shown in the 2-D diffraction pattern (insets of Figure 2d), can be simply fitted using a Gaussian function:

$$I_A(q) = I_0(q) + \frac{A}{w\sqrt{\pi/2}} e^{-2\frac{(q-q_c)^2}{w^2}} \quad (8)$$

where I_0 is the offset, q_c is the center, w is the width, and A is the area.

As shown in Figure 2d, only the left section of the amorphous hump from 1.0 to 1.46 Å^{-1} was employed for the fitting since the right section was overlapped by the Bragg peaks. The data were then fitted using OriginPro 2017. For both the cases of the pristine and the deswelled samples, the Gaussian fitting was successful, as evidenced by the values of R-squared > 99.9%.

The profile of $I_C(q)$ can be obtained by subtracting $I_A(q)$ from $I(q)$. Here we only compared the value of the full width at half maximum (FWHM) of the Bragg peak centered at 1.53 Å^{-1} to estimate the change of the crystallite size after PNP treatment. The Bragg peaks were further fitted by Gaussian functions for q ranging from 1.44 to 1.58 Å^{-1} (Supplementary Figure 7). The FWHM values for the peaks from the pristine and the modified samples are 0.032 and 0.040 Å^{-1}, respectively.

Characterization of membrane transport properties in RO

Membrane transport properties, i.e., water and salt permeabilities, were determined in a lab-scale crossflow RO unit[5]. The effective membrane area available for permeation was 20.02 cm^2 (7.7 cm × 2.6 cm), the crossflow velocity was fixed at 21.4 cm s^{-1}, and the temperature was 25 ± 0.2 °C. The loaded membrane was first compacted with DI water at an applied pressure of 31 bar (450 psi) until the permeate flux reached steady state. The applied pressure was then decreased to 27.6 bar (400 psi) to determine the pure water flux. The water permeability coefficient, A, was then calculated from:

$$A = \frac{J_{\mathrm{w}}}{\Delta P} \quad (9)$$

where J_{w} is the measured pure water flux, and ΔP is the applied pressure. Salt rejection was determined at 27.6 bar (400 psi) using a calibrated conductivity meter (Oakton Instruments, USA). The observed NaCl rejection, R, was calculated from:

$$R = 1 - \frac{C_{\mathrm{p}}}{C_{\mathrm{f}}} \quad (10)$$

where C_{p} is the permeate NaCl concentration and C_{f} is the feed NaCl concentration. The salt permeability coefficient, B, was determined from[6,7]:

$$B = J_{\mathrm{w}}^{\mathrm{NaCl}} \frac{1-R}{R} \exp\left(-\frac{J_{\mathrm{w}}^{\mathrm{RO}}}{k}\right) \quad (11)$$

where $J_{\mathrm{w}}^{\mathrm{NaCl}}$ is the measured water flux with NaCl feed solution, k is the mass transfer coefficient in the crossflow cell, which can be obtained from a correlation with rectangular channel geometry in laminar flow:

$$Sh = \frac{kd_{\mathrm{h}}}{D} = 1.85\left(\frac{R_e S_c d_{\mathrm{h}}}{L}\right)^{0.33} \quad (12)$$

where D is the NaCl diffusion coefficient, R_e is the Reynolds number, S_c is the Schmidt number, d_{h} is the hydraulic diameter, and L is the length of the channel.

Supplementary Discussion

Analysis of hydrogen bond interaction between p-nitrophenol with cellulose triacetate

The interaction of p-nitrophenol (PNP) with cellulose triacetate (CTA) material is important in determining the effect of plasticization. Specifically, CTA polymer is abundant in oxygen-containing groups, such as carbonyl (C=O) and hydroxyl groups (C−OH), which could serve as hydrogen-bond acceptors. In the molecular structure of PNP, the nitro group in the para position of the aromatic ring tends to withdraw electrons, thereby reducing the electron density of the phenolic hydroxyl group to serve as a hydrogen-bond donor. As such, the hydrogen atom of the phenolic hydroxyl of PNP will favorably form a hydrogen bond with oxygen-containing groups on the CTA chain, thus facilitating the penetration of PNP into the CTA polymer matrix to induce the plasticizing effect. To verify the presence of hydrogen bonding, we characterized the fabricated CTA films using Fourier transform infrared (FTIR) and the data are presented in Supplementary Figure 4.

The pristine CTA film (black curve) displays intensified peaks at 1747, 1232, and 1047 cm^{-1}, which are ascribed to the oxygen-containing groups including C=O, C−O, and C−O−C, respectively[8]. After treating with PNP, the swelled film (red curve) exhibited three new peaks at 1593, 1502, and 1340 cm^{-1} (labelled with purple text). The peak at 1593 cm^{-1} is assigned to the stretch of C=C on the aromatic ring and the peaks at 1502 and 1340 cm^{-1} arise from the stretch of the nitro group[9]. Notably, the presence of PNP led to the red shift of the peaks to lower wavenumbers for all the oxygen-containing groups. For example, the peak of C=O shifts to 1731 cm^{-1}. These red shifts are likely ascribed to formation of hydrogen bonds between the hydroxyl group of PNP with the oxygen-containing groups of CTA, thereby inhibiting the stretch of these groups to lower frequencies[10,11]. After rinsing with water, the characteristic peaks of PNP disappeared on the deswelled film (blue curve), consistent with the release of PNP observed in other measurements (i.e., POM, DSC, and WAXD). Notably, the peaks of oxygen-containing groups also recovered to their corresponding positions and shape in the pristine samples. Therefore, these results provide further support for the plasticizing-extracting mechanism. Additionally, forming hydrogen bonding requires the hydrogen donor group in its protonated form rather than deprotonated form, which could explain the ineffectiveness of the deprotonated PNP in modifying CTA membranes (Supplementary Figures 2 and 3).

Analysis of the effect of membrane transport properties on desalination performance

(1) Analysis of water flux

In osmotically-driven membrane processes, water flux, J_w, across the membrane is determined by:

$$J_\mathrm{w} = A\Delta\pi_\mathrm{m} \tag{13}$$

where A is the water permeability, $\Delta\pi_\mathrm{m}$ is the effective osmotic pressure across the active layer of the membrane.

Due to the detrimental effects of external concentration polarization (ECP) in the draw solution, internal concentration polarization (ICP) within the porous support, and reverse salt flux, J_s, across the membrane, the $\Delta\pi_\mathrm{m}$ is lower than the osmotic pressure difference between the bulk draw and feed solutions and could be determined by[12–14]:

$$\Delta\pi_m = \frac{\pi_{D,b}\exp\left(-\frac{J_w}{k}\right) - \pi_{F,b}\exp\left(\frac{J_w S}{D}\right)}{1 + \frac{B}{J_w}\left[\exp\left(\frac{J_w S}{D}\right) - \exp\left(-\frac{J_w}{k}\right)\right]} \tag{14}$$

where $\pi_\mathrm{D,b}$ and $\pi_\mathrm{F,b}$ are the osmotic pressures of the bulk draw and feed solutions, respectively, k is the mass transfer coefficient of ECP, S is the structural parameter of the membrane support layer, and D is the diffusion coefficient of the solute.

In Supplementary Equation 14, the terms, $\exp(J_\mathrm{w}S/D)$ and $\exp(-J_\mathrm{w}/k)$, are the detrimental effects of ICP and ECP, respectively. The term in the denominator, $1+B/J_\mathrm{w}[\exp(J_\mathrm{w}S/D)-\exp(-J_\mathrm{w}/k)]$, is the detrimental effect of reverse salt flux. The water flux could be calculated by substituting Supplementary Equation 14 into Supplementary Equation 13:

$$J_\mathrm{w} = A\left\{\frac{\pi_\mathrm{D,b}\exp\left(-\frac{J_\mathrm{w}}{k}\right) - \pi_\mathrm{F,b}\exp\left(\frac{J_\mathrm{w} S}{D}\right)}{1 + \frac{B}{J_\mathrm{w}}\left[\exp\left(\frac{J_\mathrm{w} S}{D}\right) - \exp\left(-\frac{J_\mathrm{w}}{k}\right)\right]}\right\} \tag{15}$$

We rephrase Supplementary Equation 15 by moving A to the denominator:

$$J_\mathrm{w} = \left\{\frac{\pi_\mathrm{D,b}\exp\left(-\frac{J_\mathrm{w}}{k}\right) - \pi_\mathrm{F,b}\exp\left(\frac{J_\mathrm{w} S}{D}\right)}{\frac{1}{A} + \frac{B}{A}\frac{1}{J_\mathrm{w}}\left[\exp\left(\frac{J_\mathrm{w} S}{D}\right) - \exp\left(-\frac{J_\mathrm{w}}{k}\right)\right]}\right\} \tag{16}$$

In the denominator, the decrease of A value through PNP treatment led to a larger value in term $1/A$. On the other hand, the enhanced water/salt selectivity (i.e., larger A/B value or smaller B/A value) gave a smaller value in the second term, $(B/A)(1/J_w)[\exp(J_w S/D)-\exp(-J_w/k)]$, thereby offsetting the increase in the term $1/A$ to keep the denominator constant. Generally, the overall performance of J_w remained unchanged under the operational conditions in our study.

(2) Analysis of reverse salt flux

In osmotically-driven membrane processes, reverse salt flux, J_s, across the membrane is determined by:

$$J_s = B\Delta C_m \tag{17}$$

where B is the salt permeability, ΔC_m is the effective concentration gradient across the active layer of the membrane, which could be determined by: [12, 13, 14]

$$\Delta C_m = \frac{C_{D,b}\exp\left(-\frac{J_w}{k}\right) - C_{F,b}\exp\left(\frac{J_w S}{D}\right)}{1+\frac{B}{J_w}\left[\exp\left(\frac{J_w S}{D}\right)-\exp\left(-\frac{J_w}{k}\right)\right]} \tag{18}$$

where $C_{D,b}$ and $C_{F,b}$ are the concentrations of the bulk draw and feed solutions, respectively. The reverse salt flux could be calculated by substituting Supplementary Equation 18 into Supplementary Equation 17:

$$J_s = B\left\{\frac{C_{D,b}\exp\left(-\frac{J_w}{k}\right) - C_{F,b}\exp\left(\frac{J_w S}{D}\right)}{1+\frac{B}{J_w}\left[\exp\left(\frac{J_w S}{D}\right)-\exp\left(-\frac{J_w}{k}\right)\right]}\right\} \tag{19}$$

We rephrase Supplementary Equation 19 by moving B to the denominator:

$$J_s = \left\{\frac{C_{D,b}\exp\left(-\frac{J_w}{k}\right) - C_{F,b}\exp\left(\frac{J_w S}{D}\right)}{\frac{1}{B}+\frac{1}{J_w}\left[\exp\left(\frac{J_w S}{D}\right)-\exp\left(-\frac{J_w}{k}\right)\right]}\right\} \tag{20}$$

In the denominator, the decrease of B value through PNP treatment led to a larger value in term $1/B$, thereby resulting in a larger value in the denominator that gave a decreased J_s.

In summary, the water flux is affected by both water and salt permselectivities, whereas the reverse salt flux is mainly determined by the salt permeability. Therefore, we observed unchanged water flux with decreased reverse salt flux for the membranes treated with low concentrations of PNP solutions.

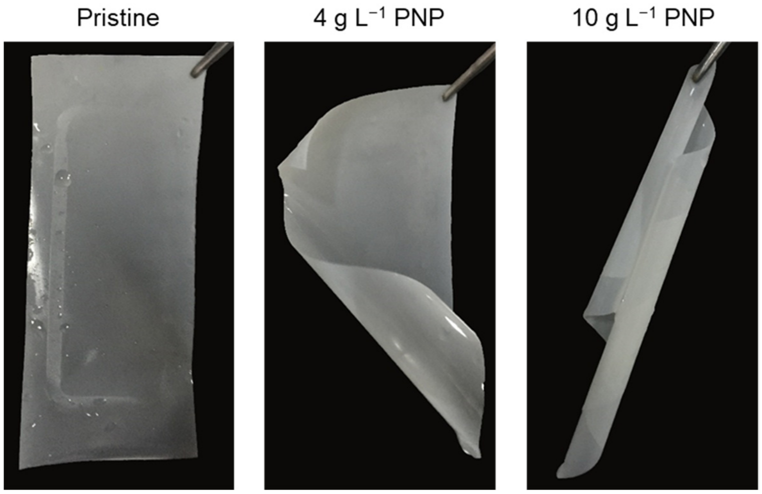

Supplementary Figure 1. Photos of pristine, 4 g L^{-1}, and 10 g L^{-1} PNP modified membranes. These membranes correspond to the membranes with desalination performance reported in Figure 1b. The modified membranes exhibit curling toward the active layer, with curling becoming more pronounced with increasing PNP concentration from 4 to 10 g L^{-1}.

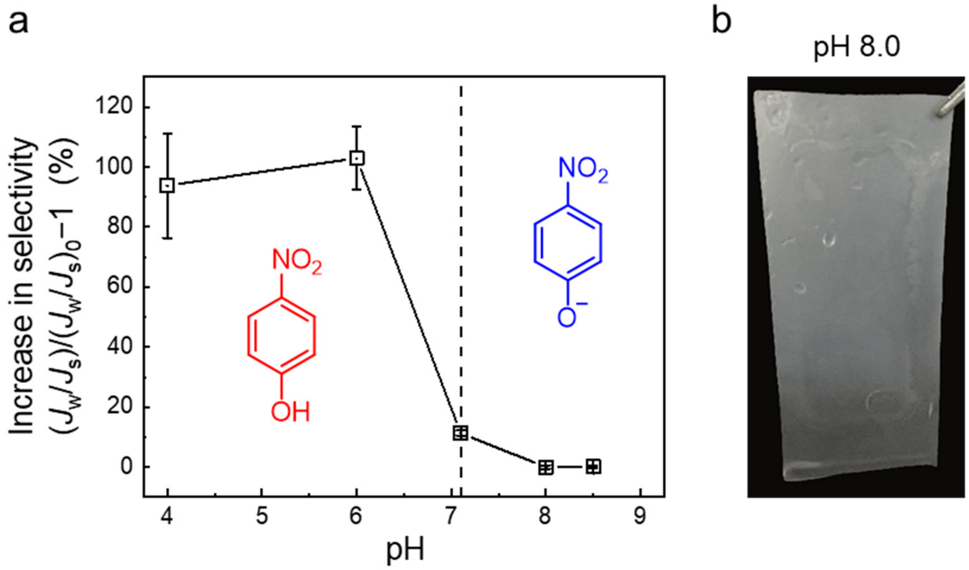

Supplementary Figure 2. (a) Effect of PNP solution pH on the water/salt selectivity of modified membranes. The dashed line indicates the pK_a 7.1 of PNP. Results are presented as percentage increase in selectivity relative to that of the pristine membranes. Error bars represent standard deviation from duplicate experiments. (b) Photos of the membrane soaked in 4 g L^{-1} PNP at pH 8.0. Curling was not observed when PNP did not alter the transport properties of the membrane.

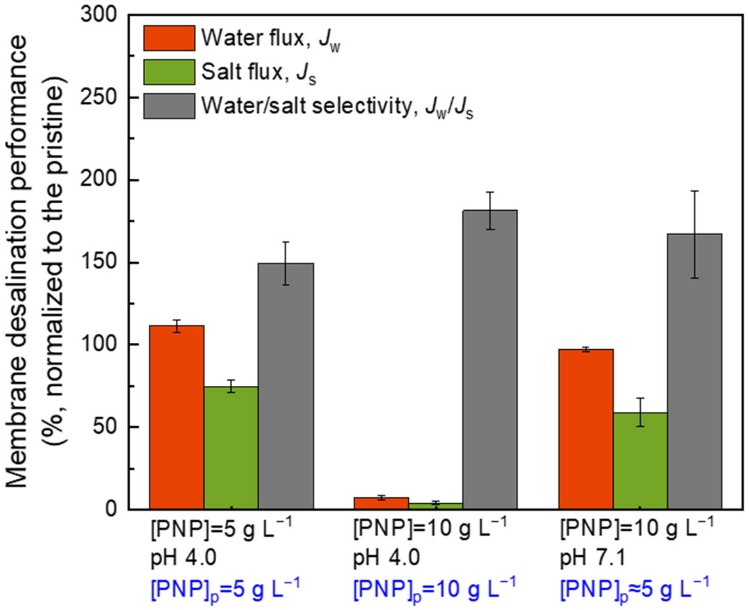

Supplementary Figure 3. Influence of effective concentration of the protonated PNP on the desalination performance and water/salt selectivity of the CTA membrane. The pK_a of PNP is 7.1. At pH 4.0, the protonated form of PNP is the dominant species; that is, the concentration of the protonated PNP (i.e., $[PNP]_p$) is equal to the concentration of total PNP (i.e., [PNP]) (left and middle panels). When pH is around the pK_a 7.1, the protonated form accounts for about half of the total PNP (right panel). All the performance data are normalized to that of the pristine membranes. Error bars represent standard deviation from duplicate experiments.

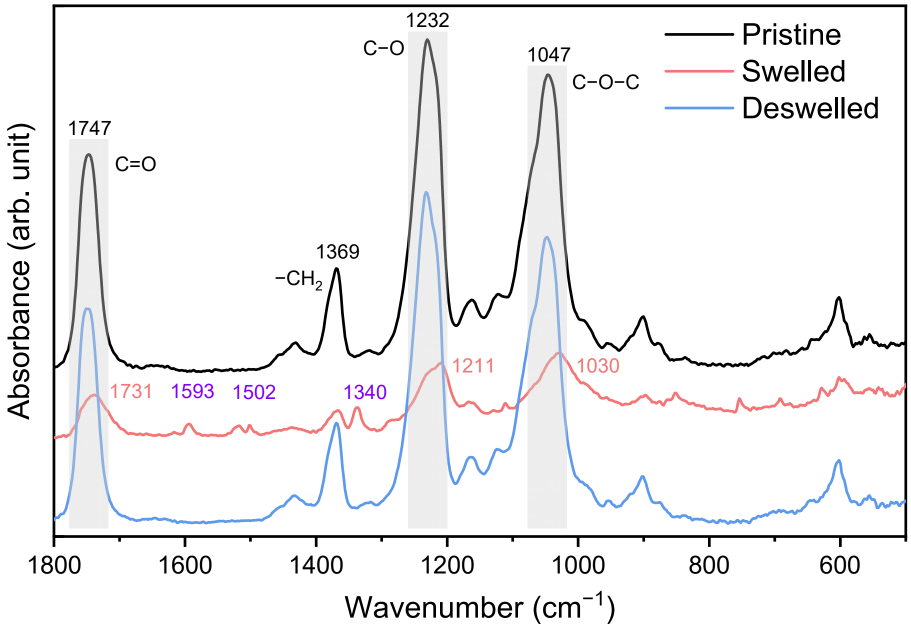

Supplementary Figure 4. Fourier transform infrared (FTIR) spectra for the pristine and modified CTA films. Measurements were carried out on air-dried samples with 64 scans.

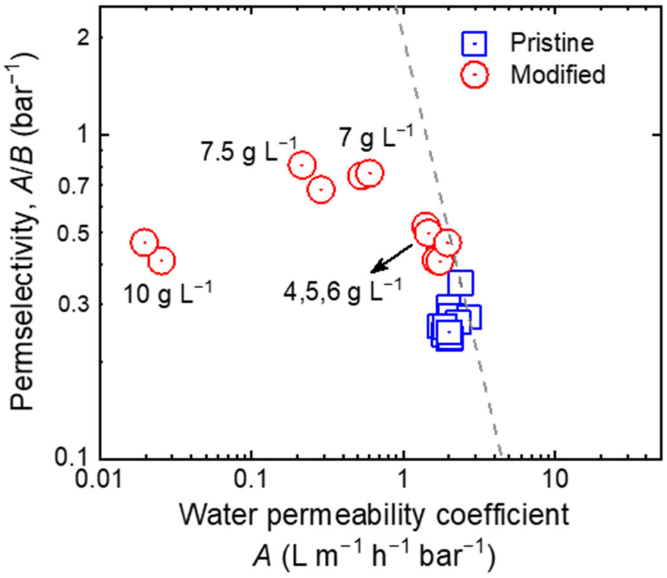

Supplementary Figure 5. Correlation between water/salt permselectivity, A/B, and water permeability coefficient, A, of membranes modified at different PNP concentrations. Blue squares and red circles represent the measured data of pristine and modified membranes, respectively. The dashed line represents the proposed upper bound relationship between water/salt permselectivity and water permeability coefficients where $A/B = \lambda/A^{\beta}$ with $\lambda = 2.0$ and $\beta = 2.0$.

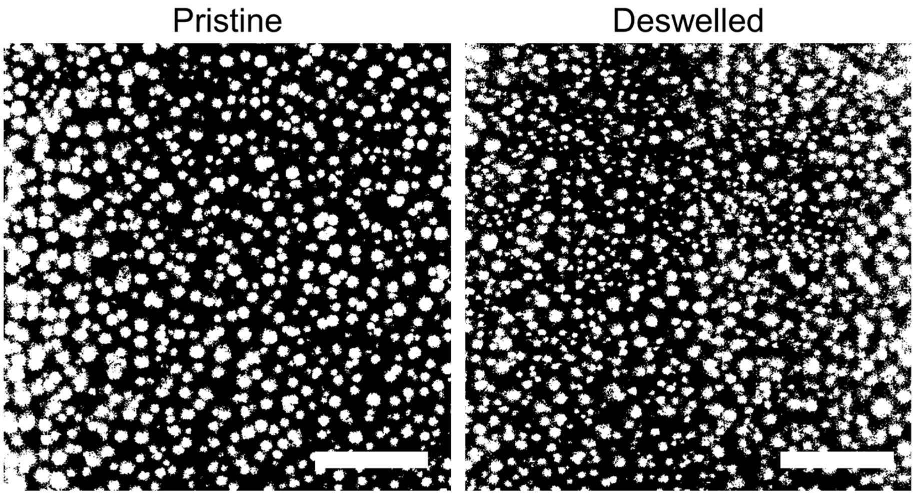

Supplementary Figure 6. Artificial coloring to highlight the birefringent domains in the POM images. The original POM images with a size of 0.8 mm × 0.8 mm were converted to black and white ones by setting a threshold. Pixels in the image whose values lie under the threshold are converted to black and pixels with values above the threshold are converted to white. The birefringent domains in the deswelled film are smaller than those in the pristine film, suggesting the decrease of crystallite size after PNP treatment. Scale bar, 200 μm.

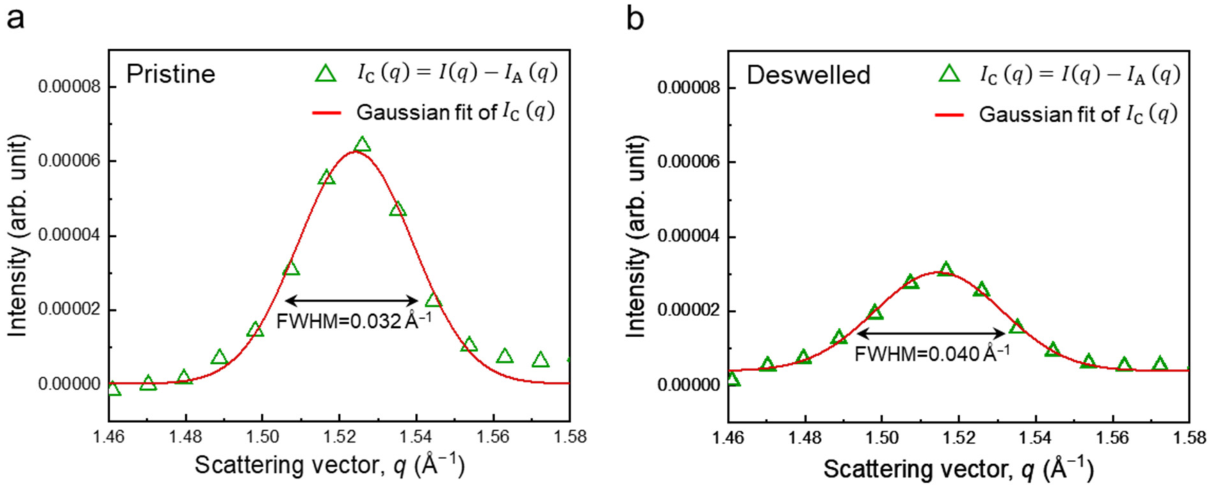

Supplementary Figure 7. Analysis of the 1-D X-ray diffraction data of pristine (a) and deswelled (b) CTA films after subtracting the amorphous component. The experimental data (green triangles) were fitted using Gaussian functions (red lines).

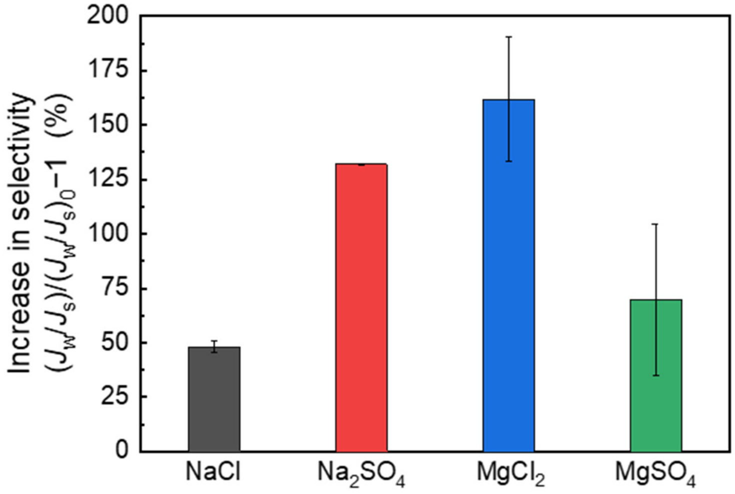

Supplementary Figure 8. Increase in selectivity of different salts using the modified CTA membrane. Pristine CTA membrane was soaked in 5 g L^{-1} PNP solution at pH 4.0 for 0.5 h and then rinsed with water for 8 h. Desalination performance was determined using 1 mol L^{-1} salt draw solution and DI water feed solution with membrane active layer facing the draw solution. Results are presented as percentage increase in selectivity relative to that of the pristine membranes. Error bars represent standard deviation from duplicate experiments.

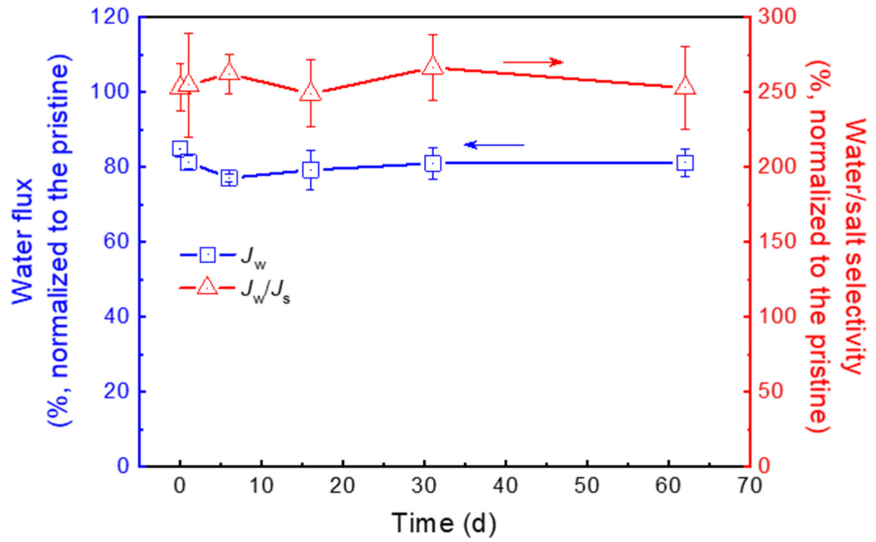

Supplementary Figure 9. Long-term desalination performance of the modified CTA membrane. Pristine CTA membrane was soaked in 4 g L^{-1} PNP solution at pH 4.0 for 18 h and then rinsed with water for 8 h to achieve the modified membrane. All the performance data are normalized to that of the pristine membranes. Error bars represent standard deviation from duplicate experiments. The modified membrane showed stable performance after 61 days, indicative of an irreversible effect induced by PNP treatment.

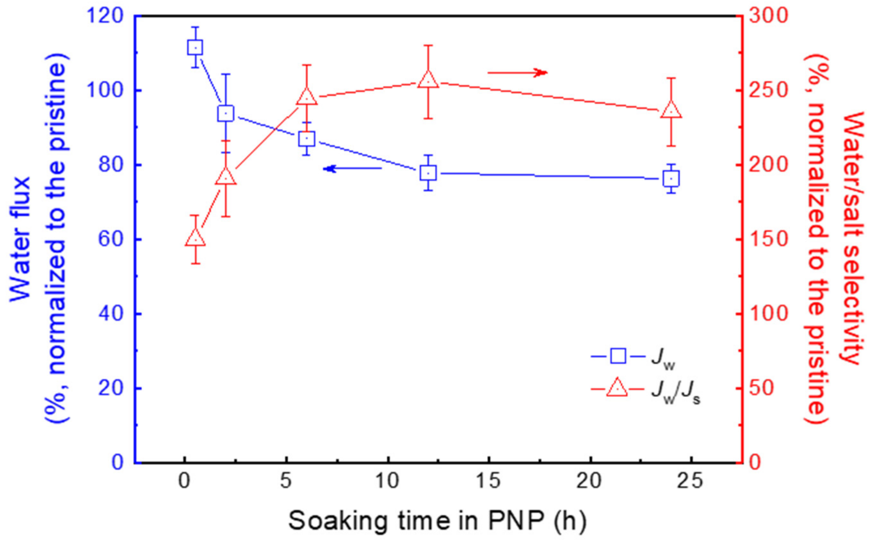

Supplementary Figure 10. Effect of soaking time in PNP solution on water flux and water/salt selectivity of the polymeric CTA membrane. A pristine CTA membrane was soaked in 5 g L^{-1} PNP solution at pH 4.0 for various soaking times and then rinsed with water for eight hours. All the performance data are normalized to that of the pristine membranes. Error bars represent standard deviation from duplicate experiments.

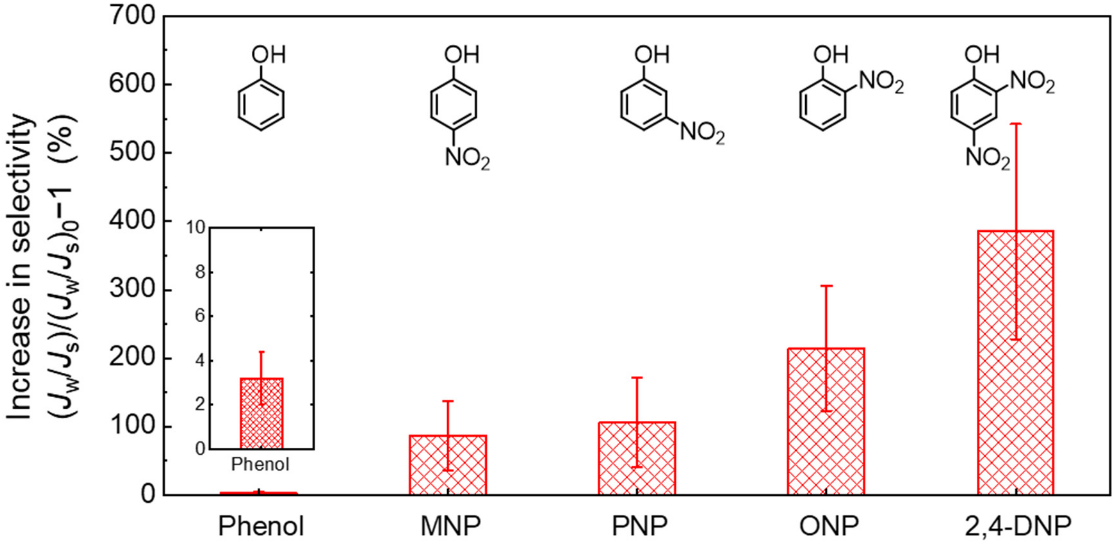

Supplementary Figure 11. Comparison of the increase in selectivity for polymeric CTA membranes soaked in different phenolic solutions. Pristine CTA membrane coupons were soaked in 4 g L^{-1} phenol, m-nitrophenol (MNP), p-nitrophenol (PNP), o-nitrophenol (ONP), or 2,4-dinitrophenol (2,4-DNP) at pH 4 for 0.5 h. The soaked membranes were subsequently rinsed with water for 8 h. Results are presented as percentage increase in selectivity relative to that of the pristine membranes. Error bars represent standard deviation from duplicate experiments.

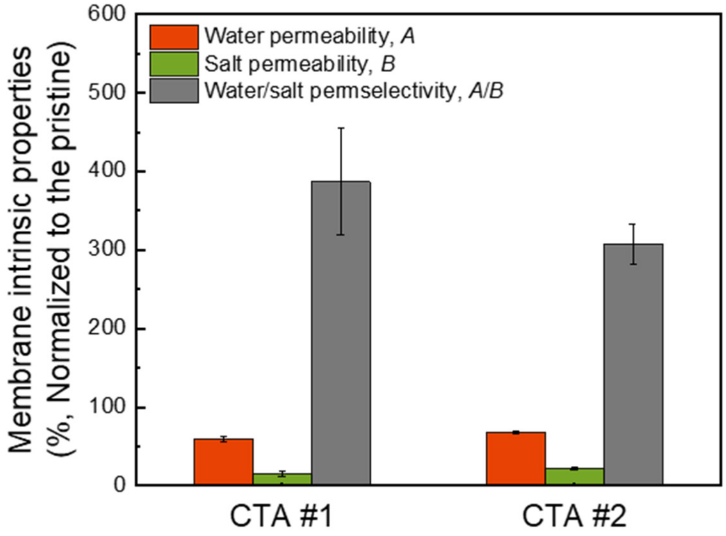

Supplementary Figure 12. Transport properties of two types of CTA membranes determined in RO. The test was conducted in a lab-scale RO setup at 27.6 bar, using 50 mM NaCl as a feed solution at 25 °C. Pristine membranes were soaked in 6 g L^{-1} PNP solution at pH 4.0 for 0.5 h, followed by water rinsing for 8 h to obtain modified samples. Transport properties of a modified membrane were normalized to those of its corresponding pristine sample. Error bars represent standard deviation from duplicate experiments. The modified membranes displayed decreases in both water permeability, *A*, and salt permeability, *B*. Notably, the decrease of salt permeability is more pronounced than that of water permeability, thereby leading to the remarkable increase in permselectivity (i.e., *A*/*B*) after PNP modification. Taken together, these results imply that the effect of PNP treatment on transport properties is also effective in RO.

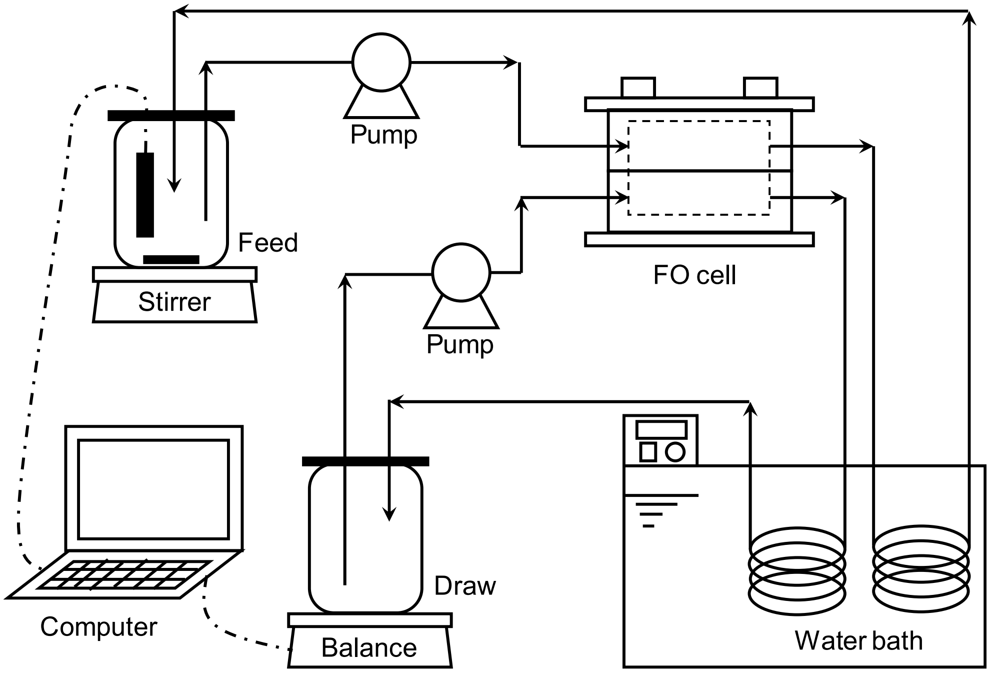

Supplementary Figure 13. Schematic of the custom-built FO setup used to determine membrane desalination performance. Unless otherwise specified, experiments were conducted under the following conditions: 1.0 M NaCl draw solution, DI water feed solution, temperature of 25 °C, crossflow velocity of 16 cm s^{-1}, with the active layer facing draw solution.

Supplementary Table 1. Desalination performance and transport coefficients for pristine and modified CTA membranes. [a]

Membrane	Pristine							Modified								
	J_w	J_s	Pe^s	Pe^δ	A	B	A/B	C_{PNP}	J_w	J_s	Pe^s	Pe^δ	A	B	A/B
	$L\ m^{-2}\ h^{-1}$	$mol\ m^{-2}\ h^{-1}$	-	-	$L\ m^{-2}\ h^{-1}\ bar^{-1}$	$L\ m^{-2}\ h^{-1}$	bar^{-1}	$g\ L^{-1}$	$L\ m^{-2}\ h^{-1}$	$mol\ m^{-2}\ h^{-1}$	-	-	$L\ m^{-2}\ h^{-1}\ bar^{-1}$	$L\ m^{-2}\ h^{-1}$	bar^{-1}
#1	13.53	1.15	1.51	0.20	1.99	8.39	0.24	4	14.07	0.62	1.57	0.20	1.73	3.81	0.46
#2	13.23	1.04	1.47	0.19	1.76	6.85	0.26	4	13.28	0.51	1.48	0.19	1.41	2.70	0.52
#3	15.01	0.87	1.67	0.22	2.38	6.84	0.35	5	13.71	0.67	1.53	0.20	1.65	4.01	0.41
#4	13.97	0.96	1.56	0.20	1.99	6.79	0.29	5	13.95	0.69	1.55	0.20	1.74	4.28	0.41
#5	13.30	1.11	1.48	0.19	1.85	7.61	0.24	6	13.38	0.54	1.49	0.19	1.46	2.93	0.50
#6	13.96	1.02	1.55	0.20	2.05	7.40	0.28	6	14.63	0.64	1.63	0.21	1.96	4.21	0.46
#7	14.95	1.10	1.67	0.22	2.70	9.89	0.27	7	9.04	0.24	1.01	0.13	0.53	0.71	0.75
#8	14.28	1.09	1.59	0.21	2.30	8.71	0.26	7	9.60	0.25	1.07	0.14	0.60	0.79	0.76
#9	13.53	1.17	1.51	0.20	2.01	8.57	0.23	7.5	6.58	0.20	0.73	0.10	0.29	0.42	0.68
#10	13.48	1.06	1.50	0.19	1.88	7.35	0.26	7.5	5.59	0.14	0.62	0.08	0.22	0.27	0.81
#11	13.61	1.16	1.52	0.20	2.04	8.64	0.24	10	1.11	0.05	0.12	0.02	0.03	0.06	0.41
#12	13.64	1.12	1.52	0.20	2.01	8.16	0.25	10	0.88	0.04	0.10	0.01	0.02	0.04	0.47

[a] Desalination performance was determined using 1 mol L^{-1} NaCl draw solution and DI water feed solution with membrane active layer facing the draw solution.

Supplementary Table 2. Effect of p-nitrophenol (PNP) treatment on desalination performance of two types of CTA membranes. [a]

Membrane	Pristine [b]			Modified [c]			Increase in selectivity [d]
	Water flux, J_w ($L\ m^{-2}\ h^{-1}$)	Salt flux, J_s ($mol\ m^{-2}\ h^{-1}$)	Selectivity, J_w/J_s ($L\ mol^{-1}$)	Water flux, J_w ($L\ m^{-2}\ h^{-1}$)	Salt flux, J_s ($mol\ m^{-2}\ h^{-1}$)	Selectivity, J_w/J_s ($L\ mol^{-1}$)	$(J_w/J_s)/(J_w/J_s)_0-1$ (%)
**CTA loose**	14.49 ± 0.74	0.91 ± 0.01	15.92 ± 1.93	11.05 ± 0.01	0.30 ± 0.01	37.36 ± 1.63	136 ± 18
**CTA dense**	13.48 ± 0.59	0.13 ± 0.02	108.81 ± 16.37	14.81 ± 0.23	0.08 ± 0.01	180.19 ± 26.12	66 ± 1

[a] Desalination performance was determined using 1 mol L^{-1} NaCl draw solution and DI water feed solution with membrane active layer facing the draw solution. [b] The dense membrane exhibited higher selectivity compared to the loose membranes. [c] Membranes were treated in 4 g L^{-1} PNP solution at pH 4.0 for 18 h and subsequently rinsed with water for 8 h. [d] PNP treatment lead to increased selectivity for both membranes.

.

Supplementary References

1. Yong JS, Phillip WA, Elimelech M. Coupled reverse draw solute permeation and water flux in forward osmosis with neutral draw solutes. *Journal of Membrane Science* **392–393**, 9-17 (2012).
2. Yip NY, Tiraferri A, Phillip WA, Schiffman JD, Elimelech M. High Performance Thin-Film Composite Forward Osmosis Membrane. *Environmental Science & Technology* **44**, 3812-3818 (2010).
3. Hancock NT, Cath TY. Solute Coupled Diffusion in Osmotically Driven Membrane Processes. *Environmental Science & Technology* **43**, 6769-6775 (2009).
4. Phillip WA, Yong JS, Elimelech M. Reverse Draw Solute Permeation in Forward Osmosis: Modeling and Experiments. *Environmental Science & Technology* **44**, 5170-5176 (2010).
5. Lu X, Boo C, Ma J, Elimelech M. Bidirectional Diffusion of Ammonium and Sodium Cations in Forward Osmosis: Role of Membrane Active Layer Surface Chemistry and Charge. *Environmental Science & Technology* **48**, 14369-14376 (2014).
6. Baker RW. *Membrane Technology and Applications*, 2nd edn (John Wiley & Sons, Ltd, 2004).
7. Mulder M. *Basic Principles of Membrane Technology*, 3rd edn (Springer Science & Business Media, 2012).
8. Luo W, Xie M, Hai FI, Price WE, Nghiem LD. Biodegradation of cellulose triacetate and polyamide forward osmosis membranes in an activated sludge bioreactor: Observations and implications. *Journal of Membrane Science* **510**, 284-292 (2016).
9. Abkowicz-Bieńko AJ, Latajka Z, Bieńko DC, Michalska D. Theoretical infrared spectrum and revised assignment for para-nitrophenol. Density functional theory studies. *Chemical Physics* **250**, 123-129 (1999).
10. Coleman MM, Skrovanek DJ, Hu J, Painter PC. Hydrogen bonding in polymer blends. 1. FTIR studies of urethane-ether blends. *Macromolecules* **21**, 59-65 (1988).
11. Rubtsov IV, Kumar K, Hochstrasser RM. Dual-frequency 2D IR photon echo of a hydrogen bond. *Chemical Physics Letters* **402**, 439-443 (2005).
12. Yip NY, Elimelech M. Performance Limiting Effects in Power Generation from Salinity Gradients by Pressure Retarded Osmosis. *Environmental Science & Technology* **45**, 10273-10282 (2011).
13. Yip NY*, et al.* Thin-film composite pressure retarded osmosis membranes for sustainable power generation from salinity gradients. *Environmental Science & Technology* **45**, 4360-4369 (2011).
14. Tiraferri A, Yip NY, Straub AP, Romero-Vargas Castrillon S, Elimelech M. A method for the simultaneous determination of transport and structural parameters of forward osmosis membranes. *Journal of Membrane Science* **444**, 523-538 (2013).
